# Effects of Mixed Baculovirus Infections in Biological Control: A Comprehensive Historical and Technical Analysis

**DOI:** 10.3390/v15091838

**Published:** 2023-08-30

**Authors:** María Leticia Ferrelli, Ricardo Salvador

**Affiliations:** 1 Instituto de Biotecnología y Biología Molecular (IBBM, UNLP-CONICET), Facultad de Ciencias Exactas, Universidad Nacional de La Plata, La Plata 1900, Buenos Aires, Argentina; 2 Instituto de Microbiología y Zoología Agrícola (IMyZA), Centro de Investigaciones en Ciencias Agronómicas y Veterinarias (CICVyA), Instituto Nacional de Tecnología Agropecuaria (INTA), Nicolás Repetto y de los Reseros s/n, Hurlingham 1686, Buenos Aires, Argentina

**Keywords:** synergism, antagonism, additive effect, biocontrol, baculovirus mixture

## Abstract

Baculoviruses are insect-specific DNA viruses that have been exploited as bioinsecticides for the control of agricultural and forest pests around the world. Mixed infections with two different baculoviruses have been found in nature, infecting the same host. They have been studied to understand the biology of virus interactions, their effects on susceptible insects, and their insecticidal implications. In this work, we summarize and analyze the in vivo baculovirus co-infections reported in the literature, mainly focusing on pest biocontrol applications. We discuss the most common terms used to describe the effects of mixed infections, such as synergism, neutralism, and antagonism, and how to determine them based on host mortality. Frequently, baculovirus co-infections found in nature are caused by a combination of a nucleopolyhedrovirus and a granulovirus. Studies performed with mixed infections indicated that viral dose, larval stage, or the presence of synergistic factors in baculovirus occlusion bodies are important for the type of virus interaction. We also enumerate and discuss technical aspects to take into account in studies on mixed infections, such as statistical procedures, quantification of viral inocula, the selection of instars, and molecular methodologies for an appropriate analysis of baculovirus interaction. Several experimental infections using two different baculoviruses demonstrated increased viral mortality or a synergistic effect on the target larvae compared to single infections. This can be exploited to improve the baculovirus-killing properties of commercial formulations. In this work, we offer a current overview of baculovirus interactions in vivo and discuss their potential applications in pest control strategies.

## 1. Introduction

Naturally occurring co-infections involving two or more different species of virus are found in nature in all domains of life [1,2]. This is not surprising considering that viruses are the most abundant biological entities on Earth [3]. In co-infections, viral interactions can be viewed from the point of view of the interaction between viruses, i.e., when one virus replication is affected in some way by the presence of the other [1], or from the point of view of the host, when the co-infection provokes a different outcome in the host compared to single infections, for example, altering the host’s time to death. In insects, viral co-infections have been described [4,5], and there is special interest in those involving baculoviruses due to their insecticidal and biotechnological applications. Mixed baculovirus infections, i.e., the same larval host infected by two viruses, have been found in nature. Therefore, they have been studied in vitro and in vivo [6,7,8]. Also, mixed infections appear when a virus triggers the replication of covert infections [9]. Baculoviruses are insect-specific DNA viruses that infect arthropods, mainly within the order Lepidoptera. Due to their high insecticidal activity and host specificity, baculovirus-based bioinsecticides have been exploited for the control of agricultural and forest pests around the world [10,11]. In the baculovirus replication cycle, two different virion phenotypes can be produced: the occlusion-derived virus (ODV), which establishes primary infection in the midgut of the host, and the budded virus (BV), which mediates the spread of the virus within the host. Virions of the ODV phenotype are embedded within crystalline occlusion bodies (OBs), which protect the virions from adverse environmental conditions. When ingested, OBs are dissolved by the alkaline environment of the insect midgut, resulting in the release of ODVs, which cross the peritrophic membrane (PM) barrier and infect midgut epithelial cells [12]. In baculoviruses, two types of viruses are clearly distinguished by the morphology of the OBs: nucleopolyhedrovirus (NPV) and granulovirus (GV). The OBs produced by NPV infections are formed in the nucleus with a polyhedral shape and are referred to as polyhedra. GVs produce ovicylindrical OBs, called granules (also referred to as “capsules” in the reviewed literature), which are found at the nuclear–cytoplasmic interface via the rupture of nuclear membranes [13]. The *Baculoviridae* family (Class *Naldaviricetes*, Order *Lefavirales*) is classified into four genera: *Alphabaculovirus* and *Betabaculovirus* are lepidopteran-specific NPV and GV, respectively, *Gammabaculovirus* are NPVs that infect hymenopterans and *Deltabaculovirus* comprise dipteran-specific NPVs. Phylogenetic analysis based on amino acid sequences of core genes separates the *Alphabaculoviruses* into two lineages, Group I and Group II. The *Betabaculovirus* genus is also separated into two different clades, a and b [14,15]. In lepidopteran insects, most of the infected larvae show a symptomatic disease that ends in the host’s death, which is followed by the release of OBs into the environment. In natural conditions, insects acquire infections from food contaminated with baculoviral OBs.

In this review, we focus on the mixed infections of baculoviruses in vivo and analyze their effects on the host from the point of view of biocontrol. We review studies carried out on baculovirus interactions in vivo, focusing on the effects of viral interactions in the host and not on the effects produced by one virus due to the presence of another virus. Thus, when applied to an insect, the effect of a viral interaction can be classified as synergistic, additive, or antagonistic [16]. The term “synergy”, also referred to as “GTA” (greater than additivity), refers to the situation in which the combination of two viruses has a greater effect than the sum of the effects of individual applications. On the contrary, antagonism occurs when the mixture has a lower effect than the sum of the individual effects of the viruses applied separately. The additive effect, also known as “no-interaction”, “zero-interaction”, or “neutral”, refers to a situation in which the viral mixture has the effect of the sum of the individual effects [17,18]. This classification can be applied when one or both baculoviruses included in a mixture are infective for the host. It is important to note that the terminology used in the literature is heterogeneous regarding the effects of double infections. Terms such as “interference”, “inhibition”, or “enhancement” are frequently used, so in Section 2, we use these terms for the purposes of historical revision. Then, we aim to homogenize and re-classify the effects of different viral mixtures (Section 3).

**Figure 1:**
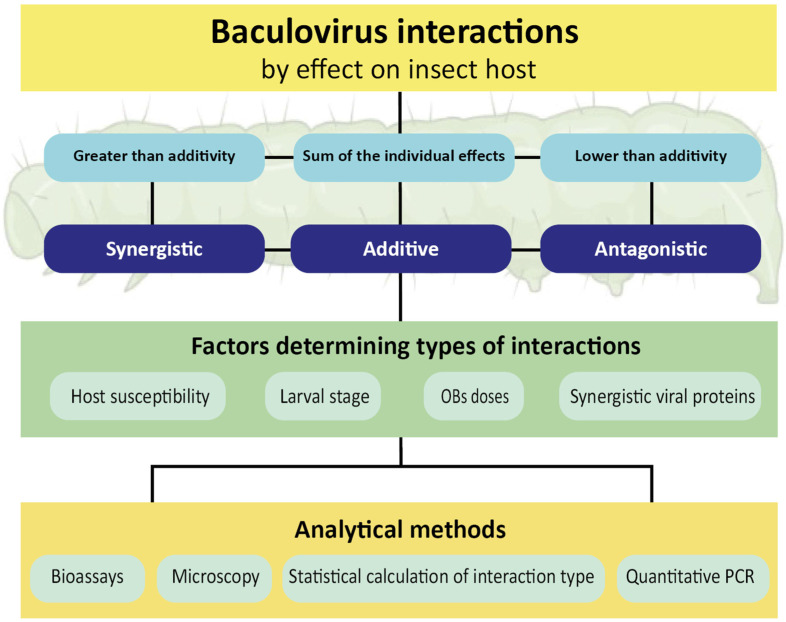
Summary of definitions, factors, and methods related with the baculovirus mixed infections in insect hosts.

Research on the in vivo co-infection of baculoviruses aims to improve their use as biopesticides. Most interaction studies have focused on mixtures of an NPV and a GV isolated from the same host species. Also, artificial baculovirus combinations have been assayed using an NPV mixed with a GV that is not infective for the host. In this study, we critically review the literature in this field and propose a classification of virus interaction based on its effect on the host. Moreover, we describe the factors that determine the type of interaction and the techniques involved in the mixed infection analysis. A schematic representation of the topics discussed here is presented in Figure 1.

## 2. Baculovirus Interactions In Vivo

### 2.1. NPV and GV Mixtures

A total of 91 baculoviral species have been registered by the International Committee on Taxonomy of Viruses [14]. Of these, only a few cases were reported infecting the same host simultaneously. The first recorded case of co-infection of a lepidopteran larva with polyhedrosis and granulosis viruses was in the cutworm *Euxoa segetum* in 1936 [19]. Similar findings were reported in *Pieris rapae*, *Pseudaletia unipuncta*, *Nephelodes emmedonia*, *Mythimna unipuncta*, *Choristoneura fumiferana*, *Spodoptera frugiperda,* and *Spodoptera Ornithogalli* [20,21,22,23,24,25]. On the other hand, the experimental co-infection with two baculoviruses isolated from the same host started with the work of Tanada [22,23]. He explored the factors influencing the susceptibility of the armyworm *Pseudaletia unipuncta* to virus infections and observed a synergistic effect when an NPV and a GV were co-inoculated into larvae. Subsequent studies aimed to characterize other co-infections involving NPVs and GVs in various larval hosts, leading to the identification of additional phenomena, such as interference and additive effects [26,27,28]. The outcomes of co-inoculating larvae with a mixture of two viruses, NPV + GV, both infectious to the host, appeared complex and difficult to predict. The different combinations of NPVs and GVs that have been reported, along with the corresponding effects observed, are presented in Table 1. For virus names, their corresponding species isolates, and genome accession numbers (if available), please refer to Table S1.

In 1959, Bird assayed different combinations of CfMNPV and CfGV in the spruce budworm *Choristoneura fumiferana* (Clemens) and found that the mortality of larvae infected with both viruses simultaneously was often higher than that of those infected with each virus individually. Interestingly, he also found that one virus interfered with the replication of the other. The author concluded that no synergistic effect was observed [26]. Nonetheless, it is important to take this analysis cautiously, as the experiments were conducted with inadequately quantified inocula at that time, preventing us from knowing the actual OB count for either virus. In subsequent studies by Lowe and Pascke [27,29,30], the quantification of viruses was improved, coupled with the introduction of bioassays. These advances allowed them to demonstrate that the mortality of double infection by NPV and GV in *Trichoplusia ni* was comparable to the mortality caused by each virus individually, concluding that no synergism or antagonism was present (referred to as interference). Also, the time to death was delayed in the double infection compared to the NPV infection [27,29]. Consequently, the overall effect on the host is compatible with an additive effect.

Whitlock (1977) found that larvae of *Heliothis armigera* (homotypic synonym: *Helicoverpa armigera*), when co-infected with both NPV and GV, exhibited an inhibitory effect compared to individual infections. Mortality rates of insects infected with both viruses were lower than those of larvae infected with a single virus [28]. Years later, Jeyarani et al. (2010) conducted an extensive experiment involving different combinations of HearGV and HearNPV across four different instars, confirming this phenomenon. They observed variable changes in mortalities, depending on the doses and instars used, with a consistent pattern of delayed death in comparison to larvae infected solely with HearNPV. Their conclusion was that HearGV did not enhance NPV infection [31]. A similar scenario arose in the case of XcenGV co-infection with XcenNPV in the larvae of the spotted cutworm *Xestia c-nigrum*. The enhancing effect was dependent on instar and dose, with XcenGV displaying a tendency to outcompete NPV infection. XcenGV enhanced XcenNPV infection in the fifth instar but not in the fourth, where the host exhibited higher susceptibility to XcenGV infection. In this instance, the GV acted as a competitor to the NPV, obscuring its enhancing action. The authors concluded that the presence and concentration of synergic factors associated with the OB play a pivotal role in viral mixtures [32]. In this way, work undertaken by Tanada, Granados, and their respective colleagues identified proteins in the occlusion bodies of TnGV and PsunGV responsible for the enhancing effect observed in certain viral mixtures (refer to Section 2.2). However, Goto (1990) was the first to demonstrate that an enhancing effect is not always observed and that the same viral mixture can show detrimental effects [32].

Based on these previous reports, Hackett (2000) measured the ‘interference’ of HzSNPV due to HearGV by assessing the survival time of *Helicoverpa zea.* It was found that HzSNPV was outcompeted by HearGV, despite the fact that HzSNPV is a fast-killing virus in contrast to HearGV. On average, higher doses of NPV caused larval death in 5.5 days, whereas in combination with HearGV at varying doses, the average killing time extended to 16.5 days. This led to the proposition that HearGV not only competed for host resources but also had an inhibitory effect on HzSNPV [18]. Similarly, Hatem et al. (2012) studied the interaction between Spodoptera littoralis NPV and GV in the cotton leafworm *S. littoralis* obtaining similar results and concluding that both viruses had an antagonistic effect. They combined different doses of SpliNPV with a constant dose of SpliGV, and vice versa. This reinforced the concept that the GV interfered with NPV replication, whereas the NPV did not hinder GV infection [33]. More recently, Wennmann et al. (2015) studied the interaction of Agrotis segetum nucleopolyhedrovirus B (AgseNPV-B) and Agrotis segetum granulovirus (AgseGV) in neonate common cutworm *Agrotis segetum* larvae. They reported that at low NPV concentrations, larvae died of granulosis, while at higher NPV concentrations, larvae mostly died with polyhedra. Based on the mortalities observed in mixed infections, they concluded that AgseGV and AgseNPV-B acted independently within the larval host, resulting in an additive interaction [34]. Conversely, bioassays performed by Cuartas-Otálora et al. (2019) demonstrated a synergistic effect when SfMNPV and SpfrGV were co-administered to *Spodoptera frugiperda* larvae. However, this synergy was observed only when the proportion of SpfrGV in the mixture did not exceed 2.5% [24]. Similar results were obtained through bioassays using SporNPV and SporGV on neonate larvae of *Spodoptera ornithogalli* [25].

The study of synergistic interactions between different baculoviruses has gained much attention due to its implications for the improvement of strategies in pest biocontrol. Following the discovery that PsunGV was responsible for the synergistic effect on PsunNPV infection, with the synergistic factor residing within the PsunGV occlusion body (formerly named ‘capsule’ and later ‘granule’) [23,35], several studies were conducted in search of the synergistic effect in various hosts and exploring viral mixtures in which only the NPV was infectious to the host and the GV, the “enhancer”.

Hukuhara and collaborators were the first to test the enhancing activity found in PsunGV in hosts that were not susceptible to PsunGV. They used it in combination with various NPVs and found that PsunGV greatly enhanced the infection of PsunNPV in *Pseudaletia separata*, slightly enhanced the infection by SpltNPV in *Spodoptera litura,* and had no effect on the infection of BmNPV in *Bombyx mori*. Therefore, they concluded that the synergistic effect varied greatly depending on the insect host and the virus used [36]. In addition to PsunGV, other GVs (as well as NPVs) were investigated for the presence of enhancing activities. Shapiro (2000) assayed the effect of HearGV and SpfrGV on LdMNPV infection in the gypsy moth *Lymantria dispar*. He found that HearGV could reduce LdMNPV LD50 by a factor of 289, while SpfrGV could reduce it 13-fold, concluding that both GVs were able to enhance LdMNPV infection [37]. Similarly, TnGV was shown to enhance the infectivity of AcMNPV in *T. ni* [38,39]. Guo and colleagues assayed XcenGV, which had previously been found to interfere with XcenNPV infection, in combination with SpltNPV in *Spodoptera litura*. In this case, where XcenGV does not naturally infect *S. litura*, they reported that the addition of XcenGV OBs significantly reduced SpltNPV LC50, producing an enhancing effect [40]. Similar findings were reported in bioassays performed by Biedma et al. (2015), where AgMNPV activity on *Anticarsia gemmatalis* larvae was enhanced by the addition of Epinotia aporema GV OBs [41]. Also here, *A. gemmatalis* was not susceptible to EpapGV. In the same vein, Jeyarani et al. (2012) assayed five GVs in combination with HearNPV across various instars of the cotton bollworm, *H. armigera.* They tested GVs from: *Spodoptera litura* (SpltGV), *Agrotis segetum* (AgseGV), *Plutella xylostella* (PlxyGV), *Achaea janata* (AjGV), and *Chilo infuscatellus* (CiGV). Among these, only SpltGV synergized with HearNPV infection, while the remaining GVs had a neutral effect [42] (Table 1).

### 2.2. Synergistic Factors

Tanada and Hukuhara (1971) demonstrated that the synergistic factor in PsunGV was a protein present in the occlusion body, often referred to as the “capsule”. On the other hand, heat inactivation treatments with HearGV revealed that when an interferent effect occurred, it was related to the virion rather than the capsule [28,31,42]. Moreover, when HearGV was introduced into a non-susceptible host, its enhancing capacity was showcased, driven by the enhancing factors within the OB [37]. Therefore, in order to avoid the inhibitory effects of the GV, one strategy is to prepare GVP (granulovirus proteins). This is achieved through a protocol that involves dissolving the OBs, eliminating the virion, and retaining the protein fraction from the OB. Experiments using an OB protein extract containing the so-called “enhancins” were conducted before and after the discovery of the *enhancin* genes (Table 2). Several studies have employed NPVs with extracts of GV OBs, as the proteins possessing enhancing activity do not need further purification. GVP obtained from XcenGV were successfully used as additives for MbMNPV on *Mamestra brassicae*, *Helicoverpa armigera*, and *Autographa nigrisigna* larvae [43,44,45,46,47].

The term “Synergistic Factor” (SF) was initially coined to describe proteins associated with the OBs of GVs that enhanced the infectivity of other baculoviruses [48,49]. These proteins were identified through bioassays involving mixed infections of PsunNPV + PsunGV. Subsequent research revealed analogous proteins referred to as “viral enhancing factors (VEFs)” or “enhancins” within the OBs of Trichoplusia ni GV (TnGV) and Xestia c-nigrum GV (XecnGV) [32,38,50]. To date, enhancin genes have been identified in both GVs and NPVs (Table 1). The analysis of sequenced baculovirus genomes indicates that *enhancin* genes are more prevalent in GVs compared to NPVs. Interestingly, the majority of GVs with reported enhancement of NPV infections belong to the phylogenetic clade “a” [15] and encode one or more *enhancin* genes in their genomes.

The function of baculovirus enhancins was studied in bioassays using intact OBs or protein extracts containing these proteins. Other studies were performed with the expression and purification of enhancins in heterologous systems [51]. The outcomes revealed two primary functions of baculovirus enhancins that influence viral potency. Wang and Granados (1997) demonstrated that it increases the permeability of the peritrophic membrane (PM), a protective barrier shielding gut cells against viruses, fungi, and bacteria. They provided evidence that GV enhancin degrades specific proteins within the PM, thereby facilitating the access of virions to the midgut [52]. Other studies performed with GV and NPV enhancins evidenced an increased fusion of virions to midgut cells [53,54]. These results were also supported by studies conducted in cell culture systems [55]. Moreover, analysis of enhancin location in baculoviruses revealed that NPV enhancins are commonly found in ODV envelopes, while GV enhancins are included within OBs [56,57]. Also, SpfrGV OB proteomic studies indicated that both *enhancin* genes encoded by this virus are expressed and incorporated into the OB structure ([58,59], Masson et al., unpublished results).

Table 1 reveals that several GVs were used in different combinations, with more than one NPV, and administered to different host species. The exception is TnGV, which was not used in larvae other than *T. ni*. Notably, PsunGV, AgseGV, HearGV, SpfrGV, and XcenGV were applied to both permissive and non-permissive hosts (Table 3). Interestingly, when the GV is capable of infecting the host, the outcomes often display an additive or antagonistic effect (interference is also reported), as observed with AgseGV, HearGV, and TnGV. Moreover, the synergistic effect is either absent or reported for only specific instars, as exemplified by PsunGV and XcenGV, where multiple instars were tested. In contrast, the same GVs act as synergists (or enhancers) of different NPVs when applied to non-permissive hosts. These are the cases of HearGV, PsunGV, SpfrGV, TnGV, and XcenGV. Exceptions are AgseGV and PsunGV, with neutral effects in *H. armigera* and *B. mori*, respectively. Given that enhancer activity may correlate with the presence of enhancins within GV genomes (and potentially within OBs), there might be a correlation between the GV’s enhancing ability and the number of enhancin genes encoded by each virus. In this context, AgseGV codes for one enhancin in contrast to HearGV or XcenGV, which code for four enhancins each, in agreement with their significant enhancing capabilities. This notion is reinforced by the inspection of their RP values, as when comparing HearGV with SpfrGV (encoding two enhancins) showing RPs of 286 vs. 13, respectively, in *L. dispar*.

### 2.3. NPV Mixtures

Few studies report the detection of different NPVs in natural mixed infections (Table 4). Early studies by Ritter and Tanada (1978) described the interaction of two different nucleopolyhedrovirus strains of the armyworm *P. unipuncta*, specifically the Typical NPV (TNPV) and Hypertrophy NPV (HNPV), which showed distinct signs of infection in the cells. They reported “interference or antagonism” between them, as co-inoculation of both viruses resulted in larvae infected solely with the TNPV strain and did not differ from the single TNPV infection. In addition, the number of cells infected with HNPV was significantly less than in the single infection [60]. Del Rincón-Castro and Ibarra (1995) provided evidence of co-infection with two polyhedrosis viruses, namely TnSNPV and AcMNPV, in cabbage looper larvae. They found that the mixture was 7.5 times more virulent than the AcMNPV control, therefore producing a synergistic effect [61]. This was later corroborated using isolated viruses in bioassays: the addition of TnSNPV to AcMNPV resulted in synergism [39]. Although a previous report mentioned an unknown viral factor present in OBs of TnSNPV capable of producing a slight alteration of larval PM [38], attempts to identify an enhancin gene in TnSNPV by southern blot were unsuccessful [39]. The absence of enhancin genes in the TnSNPV genome was later confirmed [62].

Cheng et al. (2005) reported that a multicapsid nucleopolyhedrovirus (ThorMNPV) and a single nucleopolyhedrovirus (ThorSNPV) were co-isolated from larvae of *Thysanoplusia orichalcea* L. When these viruses were separated and evaluated individually using bioassays, ThorMNPV showed a 271-fold higher potency than ThorSNPV against third-instar *Pseudoplusia includens*. However, when using the original mixture containing the majority of ThorSNPV and low levels of ThorMNPV, the infectivity on the host was low, similar to the infection caused by ThorSNPV alone. Conversely, when using a mixture containing the majority of ThorMNPV, the effect was similar to the one caused by ThorMNPV alone. Both viruses were capable of replicating in the same tissues, but never simultaneously in the same cell. Therefore, the authors cataloged the interaction of ThorMNPV and ThorSNPV as neutralistic [7,63]. From the insecticide perspective of this review, this observation aligns with an additive effect.

A natural viral mixture was isolated from the eastern spruce budworm (*Choristoneura fumiferana*). The Choristoneura fumiferana nucleopolyhedrovirus (CfMNPV) and the Choristoneura fumiferana defective nucleopolyhedrovirus (CfDEFNPV) are two different viruses that were detected co-infecting the insect host. The CfDEFMNPV isolate is unable to produce larval infection when administered orally, but in mixtures with CfMNPV, it gains the capability to infect the insect gut. Once in the hemocoel, CfDEFNPV synergizes with CfMNPV infection through unknown mechanisms [64]. It is plausible that the presence of an enhancin gene, as reported in CfMNPV, is involved in the ability of this virus to help CfDEFNPV access the midgut epithelium [65].

Lymantria dispar nucleopolyhedrovirus (LdMNPV) is one of the few reported NPVs to contain an *enhancin* gene. Therefore, it was evaluated in virus mixtures with different NPVs under laboratory conditions [66]. These investigations demonstrated that LdMNPV increases the potencies of HzSNPV, SeMNPV, and SfMNPV against their homologous hosts. It is worth mentioning that LdMNPV itself is not infective to any of the three host species tested in the aforementioned assays.

The generalist Autographa californica multiple nucleopolyhedrovirus (AcMNPV) and Rachiplusia nu nucleopolyhedrovirus (RanuNPV) have been documented in natural infections of the soybean caterpillar *Rachiplusia nu* (Guenée) [67,68]. In a recent study, Decker-Franco et al. (2021) analyzed the interaction between AcMNPV and RanuNPV on fourth-instar *R. nu* larvae. In laboratory bioassays, these authors compared the effects of single and mixed infections and reached the conclusion that co-infection exhibited a slightly increased insecticidal performance when compared to AcMNPV alone. However, this enhancement was not found to be statistically significant [69].

### 2.4. Unknown Synergistic Factor

Enhancin genes are absent in some GVs that have shown synergistic effects on the infectivity of NPVs [25,41]. This raises the question of which proteins, other than enhancins, could act in a similar way at the primary infection site. In this sense, specific baculovirus proteins have demonstrated enhancin-like activities. Studies conducted by Liu et al. (2019) found that the expressed and purified gp37 protein of Cydia pomonella granulovirus (CpGV) is capable of altering the PM structure of *Spodoptera exigua*. Accordingly, CpGV gp37 was shown to enhance the infectivity of AcMNPV on *S. exigua* larvae. Furthermore, it was confirmed that CpGV gp37 aided ODVs to cross the insect PM and fuse with midgut cells [70]. However, it is unclear whether this protein is included in the CpGV OB or not. The presence of gp37 was found to be associated with OBs or BVs in some baculoviruses [71,72].

EpapGV, a clade **b** GV, was shown to augment the virulence of AgMNPV in *A. gemmatalis*. As EpapGV does not encode *enhancin* genes, gp37 has been proposed to be responsible for this effect [41]. However, in subsequent proteomic assays, no gp37 was found associated with EpapGV OB [73]. Therefore, the enhancement effect could be due to a different factor present in the OB. Similarly, SporGV, which was proven to synergize the SporNPV action on *S. ornithogalli*, lacks an enhancin gene, implying that other factors are supposed to act as enhancers [25]. Also, as mentioned earlier, Lara-Reyna et al. (2003) demonstrated a synergistic effect resulting from the addition of TnSNPV to AcMNPV in *T. ni* larvae [39]. Considering the absence of an enhancin gene in TnSNPV and the fact that AcMNPV OBs were dominant in infected larvae, it is possible that the enhancing effect occurs at the entry site within the midgut by means of another factor present in TnSNPV OB.

Additional proteins have been identified for their role in the disruption of the peritrophic membrane, aiding the encounter of ODVs with target epithelial cells. Baculoviral ODV-E66 is an ODV-specific protein that is conserved in lepidopteran baculoviruses [74]. Sugiura et al. (2013) demonstrated that ODV-E66 facilitates primary infection by digestion of chondroitin sulfate in the peritrophic membrane [75]. Recent studies have shown that ODV-E66 plays an important role in the ODV’s passage across the PM during oral infection by HearNPV [76]. Other baculovirus proteins, such as chitinases, have been implicated in PM disruption. Notably, Hawtin and co-workers (1995) detected chitinases within AcMNPV OBs [77]. The inclusion of chitinase in OBs has been proposed to contribute to the disruption of the chitinous peritrophic matrix [78].

## 3. Strategies to Evaluate Combinations of NPV and GVs: Technical Aspects

Taking into account that the mixture of two viruses can produce very different outcomes, including undesired effects, from the biocontrol point of view, it is critical to have several technical considerations that will be reviewed in this section.

### 3.1. Quantification of Viral Inocula

In early studies, OB quantification was not performed properly, and they talk about mixing “volumes” and dilutions of the viruses. For example, Tanada used equal volumes of “heavy suspension virus” to test mixed NPV + GV in *P. unipuncta* [22]. In more advanced works, NPV OBs were quantified through direct counting of polyhedra using a Petroff-Hausser cell counter under a light microscope. In contrast, GV “capsules’’ were quantified based on their freeze-dried weight [30] because their small size, compared to NPV OBs, made the direct count unfeasible. Similarly, Shapiro counted LdMNPV OBs using a hemocytometer and phase contrast microscope but explained that he could not count HearGV and SpfrGV OBs due to their small size. Therefore, he used different dilutions of stock suspensions to prepare the mixtures [37]. Later, with the introduction of the bioassay technique to evaluate mixed infections [30], Lowe and Paschke used a mix of 1 LD50 unit of each virus in 2 µL of total volume for feeding larvae to reduce the possibility of giving a biological advantage to any of the viruses [27]. Nowadays, quantification of GV OBs has been improved by microscopic counting using dark field illumination with the Petroff-Hausser counter [79]. As an alternative approach, Cuartas et al. (2019) quantified SpfrGV OBs by measuring absorbance at 280 nm and extrapolating from a standard curve [24].

**Table 1:** Studies on NPV + GV combinations in larval hosts.

Host	Instar	NPV	NPV Group	GV	GV Clade	GV Infects the Host	Enhancin Genes (GV)	Effect on Time to Kill	Effect on Virulence	Relative Potency	Overall Effect (Reported)	Calculated Effect (This Work)	Reference
*P. unipuncta*	third, 4th, fifth, sixth	PsunNPV	ND	PsunGV	a	yes	3	ND	increased mortality	ND (1)	synergistic	synergistic/additive (5)	[22]
*C. fumiferana*	third, fourth	CfMNPV (6)	I	CfGV (6)	b	yes	0	not affected	increased mortality	-	interference/not synergistic	antagonistic	[26]
*T. ni*	fourth	TnNPV	II	TnGV	a	yes	3	delayed	reduced mortality	-	additive		[27]
*P. unipuncta*	fifth	PsunNPV	ND	PsunGV	a	yes	3	ND	reduced ID50	56.3 (2)	synergistic		[35]
*H. armigera*	second/third	HearNPV	II	HearGV (6)	a	yes	4	delayed	reduced mortality	-	interference	antagonistic	[28]
*P. separata*	fifth	PsunNPV		PsunGV	a	yes	3	ND	reduced LD50	15,523.3 (2, 3)	enhancement		[36]
*S. litura*	fifth	SpliNPV	II	PsunGV	a	no	3	ND	reduced LD50	11.5 (2, 3)	enhancement		[36]
*B. mori*	third	BmNPV	I	PsunGV	a	no	3	ND	no effect	-	no effect		[36]
*X. c-nigrum*	fourth, fifth	XcenNPV	ND	XcenGV	a	yes	4	ND	increased ID50/reduced ID50	0.9/240.2 (2, 3)	not enhanced/enhanced		[32]
*H. zea*	first, second	HzSNPV	II	HearGV (6)	a	yes	4	delayed	ND	-	interference		[18]
*L. dispar*	second	LdMNPV	II	HearGV (6)	a	no	4	reduced LT50	reduced LD50	286.4	enhancement		[37]
*L. dispar*	second	LdMNPV	II	SpfrGV (6)	a	no	2	not affected	reduced LD50	13.1	enhancement		[37]
*L. dispar*	second, third, fourth	LdMNPV	II	HearGV (6)	a	no	4	Reduced LT50	increased mortality	ND (1)	enhancement	synergistic/additive (5)	[80]
*T. ni*	first	AcMNPV	I	TnGV (6)	a	yes	3	ND	reduced LC50	10.7 (2)	synergistic		[39]
*S. litura*	fifth	SpltNPV	II	XcenGV	a	no	4	not affected	reduced LC50	6.48	synergistic		[40]
*H. armigera*	second, third, fourth, fifth	HearNPV	II	HearGV (6)	a	yes	4	delayed	increased/reduced	-	no enhancement	antagonistic	[31]
*S. littoralis*	third	SpliNPV	II	SpliGV	a	yes	ND	delayed	increased LD50	0.2	antagonistic		[33]
*H. armigera*	second, third, fourth, fifth	HearNPV	II	SpltGV (6)	a	no	ND	reduced	reduced LC50	13.32 (4)	synergistic		[42]
*H. armigera*	second, third, fourth, fifth	HearNPV	II	AgseGV	a	no	1	not affected	ND	-	neutral		[42]
*H. armigera*	second, third, fourth, fifth	HearNPV	II	PlxyGV (6)	a	no	0	not affected	ND	-	neutral		[42]
*H. armigera*	second, third, fourth, fifth	HearNPV	II	AjGV	ND	no	ND	not affected	ND	-	neutral		[42]
*H. armigera*	second, third, fourth, fifth	HearNPV	II	CiGV	ND	no	ND	not affected	ND	-	neutral		[42]
*A. gemmatalis*	third	AgMNPV	I	EpapGV	b	no	0	reduced ST	increased mortality	ND (1)	enhancement	synergistic	[41]
*A. segetum*	neonates	AgseNPV-B	II	AgseGV	a	yes	1	ND	not affected mortality	-	additive	additive	[34]
*S. frugiperda*	second	SfMNPV	II	SpfGrV (6)	a	yes	2	reduced MTD	reduced LC50	11.4	enhancement		[24]
*S. ornithgalli*	neonates	SporNPV	II	SporGV	a	yes	0	ND	increased mortality	3.06	synergistic	synergistic	[25]

ND, not determined; (1) data for calculation is lacking; (2) calculated in this work; (3) average; (4) RP calculated for the instar with the best enhancement result; (5) the effect depends on instar or dose; (6) the abbreviation was modified according to ICTV’s current nomenclature.

**Table 2:** Combinations of NPVs and proteins derived from GVs (GVP).

Host	NPV	GV Proteins	Effect on Time to Kill	Overall Effect (Reported)	Reference
*P. unipuncta*	PsunNPV	PsunGV	ND	enhancement	[35]
*T. ni*	AcMNPV	TnGV	ND	enhancement	[38]
*M. Brassicae*	MbMNPV	XcenGV	reduced	enhancement	[43,46]
*H. armigera*	MbMNPV	XcenGV	reduced	enhancement	[44,45]
*A. nigrisigna*	MbMNPV	XcenGV	reduced	enhancement	[47]
*S. frugiperda*	SfMNPV	SpfrGV	ND	enhancement	[24]

ND, not determined.

**Table 3:** GVs that were used in combination with different NPVs.

Host	Instar	NPV	NPV Group	GV	GV Clade	GV Infects the Host	Enhancin Genes (GV)	Effect on Time to Kill	Effect on Virulence	Relative Potency	Overall Effect (Reported)	Calculated Effect (This Work)	Reference
*A. segetum*	neonates	AgseNPV-B	II	AgseGV	a	yes	1	ND	not affected mortality	ND (1)	additive	additive	[34]
*H. armigera*	second, third, fourth, fifth	HearNPV	II	AgseGV	a	no	1	not affected	ND	-	neutral		[42]
*H. armigera*	second, third	HearNPV	II	HearGV (5)	a	yes	4	delayed	reduced mortality	-	interference	antagonistic	[28]
*H. zea*	first, second	HzSNPV	II	HearGV (5)	a	yes	4	delayed	n/d	-	interference		[18]
*H. armigera*	second, third, fourth, fifth	HearNPV	II	HearGV (5)	a	yes	4	delayed	increased/reduced	-	no enhancement	antagonistic	[31]
*L. dispar*	second	LdMNPV	II	HearGV (5)	a	no	4	reduced LT50	reduced LD50	286.4	enhancement		[37]
*L. dispar*	second, third, fourth	LdMNPV	II	HearGV (5)	a	no	4	Reduced LT50	Reduced LC50	ND (1)	increased mortality	synergistic/additive (4)	[80]
*P. unipuncta*	third, fourth, fifth, sixth	PsunNPV	ND	PsunGV	a	yes	3	ND	increased mortality	ND (1)	synergistic	synergistic/additive (4)	[22]
*P. unipuncta*	fifth	PsunNPV	ND	PsunGV	a	yes	3	ND	reduced ID50	56.3 (2)	synergistic		[35]
*P. separata*	fifth	PsunNPV		PsunGV	a	yes	3	ND	reduced LD50	15,523.3 (2, 3)	enhancement		[36]
*S. litura*	fifth	SpltNPV (5)	II	PsunGV	a	no	3	ND	reduced LD50	11.5 (2, 3)	enhancement		[36]
*B. mori*	third	BmNPV	I	PsunGV	a	no	3	ND	no effect	-	no effect		[36]
*L. dispar*	second	LdMNPV	II	SpfrGV (5)	a	no	2	not affected	reduced LD50	13.1	enhancement		[37]
*S. frugiperda*	second	SfMNPV	II	SpfrGV (5)	a	yes	2	reduced MTD	reduced LC50	11.4	enhancement		[24]
*T. ni*	fourth	TnNPV	II	TnGV	a	yes	3	delayed	reduced mortality	-	additive		[27]
*T. ni*	first	AcMNPV	I	TnGV	a	yes	3	ND	reduced LC50	10.7 (2)	synergistic		[39]
*X. c-nigrum*	fourth, fifth	XcenNPV	ND	XcenGV	a	yes	4	ND	increased ID50/reduced ID50	0.9/240.2 (2, 3)	not enhanced/enhanced		[32]
*S. litura*	fifth	SpltNPV (5)	II	XcenGV	a	no	4	not affected	reduced LC50	6.48	synergistic		[40]

ND, not determined; (1) data for calculation is lacking; (2) calculated in this work; (3) average; (4) the effect depends on instar or dose; (5) the abbreviation was modified according to ICTV’s current nomenclature.

**Table 4:** In vivo interactions between NPVs.

Host	Instar	NPV 1	NPV 1 Group	NPV 2	NPV 2 Group	NPV 2 Infects the Host	Effect on Time to Kill	Effect on Virulence	Overall Effect (Reported)	Reference
*Pseudaletia unipuncta*	fifth	TNPV (Typical typical NPV)	ND	HNPV (Hypertrophy hypertrophy NPV)	ND	yes	ND	decreased	interference	[60]
*Trichoplusia nu*	first	AcMNPV	I	TnSNPV	II	yes	ND	decreased LD50	synergistic	[39]
*Pseudoplusia includens*	third	ThorMNPV	I	ThorSNPV	II	yes	ND	no effect	neutralistic	[63]
*Choristoneura fumiferana*	ND	CfMNPV	I	CfDEFMNPV	II	yes	ND	increased *	synergistic	[65]
*Helicoverpa zea*	second	HzSNPV	II	LdMNPV	II	no	ND	decreased LC50	synergistic	[66]
*Helicoverpa zea*	second	AcMNPV	I	LdMNPV	II	no	ND	decreased LC50	synergistic	[66]
*Helicoverpa zea*	second	AfMNPV	I	LdMNPV	II	no	ND	decreased LC50	synergistic	[66]
*Helicoverpa zea*	second	GmMNPV	I	LdMNPV	II	No	ND	decreased LC50	synergistic	[66]
*Helicoverpa zea*	second	HearMNPV	II	LdMNPV	II	no	ND	decreased LC50	synergistic	[66]
*Helicoverpa zea*	second	PxMNPV	I	LdMNPV	II	no	ND	decreased LC50	synergistic	[66]
*Helicoverpa zea*	second	RoMNPV	I	LdMNPV	II	no	ND	decreased LC50	synergistic	[66]
*Spodoptera exigua*	second	SeMNPV	II	LdMNPV	II	no	ND	decreased LC50	synergistic	[66]
*Spodoptera exigua*	second	AcMNPV	I	LdMNPV	II	no	ND	decreased LC50	synergistic	[66]
*Spodoptera exigua*	second	AfMNPV	I	LdMNPV	II	no	ND	decreased LC50	synergistic	[66]
*Spodoptera exigua*	second	GmMNPV	I	LdMNPV	II	no	ND	decreased LC50	synergistic	[66]
*Spodoptera exigua*	second	HearMNPV	II	LdMNPV	II	no	ND	decreased LC50	synergistic	[66]
*Spodoptera exigua*	second	PxMNPV	I	LdMNPV	II	no	ND	decreased LC50	synergistic	[66]
*Spodoptera exigua*	second	RoMNPV	I	LdMNPV	II	No	ND	decreased LC50	synergistic	[66]
*Spodoptera frugiperda*	second	SfMNPV	II	LdMNPV	II	no	ND	decreased LC50	synergistic	[66]
*Spodoptera frugiperda*	second	AcMNPV	I	LdMNPV	II	no	ND	decreased LC50	synergistic	[66]
*Spodoptera frugiperda*	second	AfMNPV	I	LdMNPV	II	no	ND	decreased LC50	synergistic	[66]
*Spodoptera frugiperda*	second	GmMNPV	I	LdMNPV	II	no	ND	decreased LC50	synergistic	[66]
*Spodoptera frugiperda*	second	HearMNPV	II	LdMNPV	II	no	ND	decreased LC50	synergistic	[66]
*Spodoptera frugiperda*	second	PxMNPV	I	LdMNPV	II	no	ND	decreased LC50	synergistic	[66]
*Spodoptera frugiperda*	second	RoMNPV	I	LdMNPV	II	no	ND	decreased LC50	synergistic	[66]
*Rachiplusia nu*	fourth	AcMNPV	I	RanuNPV	I	yes	reduced	slightly increased mortality	ND **	[69]

ND, not determined; * Increased infectivity in per os infection; ** Additive (calculated in this study).

### 3.2. Amount of Each Virus OB in the Viral Mix

When choosing doses of each virus to test in combination, the literature lacks explicit criteria on how to select the amount or proportion of virus to use. If the objective is to observe a difference in virulence between the NPV alone and the NPV + GV treatments, the assay should be designed to produce an observable change in mortality, or LD50, at a fixed stage of the target larvae. The combinations used in the reviewed literature are varied. At first, the authors used a single mixture of equal volumes of dense suspensions of both NPV and GV [22,26,27,28,36]. Then, some researchers started using graded concentrations of NPVs combined with graded concentrations of GV [31,32,33,35,37,80]. For instance, Jeyarani (2010) tested the combinations of LD25 and LD50 of both HearGV and HearNPV with the aim of determining the lowest dose of GV that gave the maximum mortality with HearNPV in *H. armigera* [31]. In our previous study, *Anticarsia gemmatalis* larvae were treated with mixtures of AgMNPV and EpapGV, and doses were selected on the basis of the LD50 in their respective hosts. Two mixtures were defined, using two AgMNPV doses (higher and lower than the LD50) in combination with a fixed EpapGV dose chosen based on EpapGV virulence on its native host, specifically a dose resulting in 100% mortality in the last instar of *Epinotia aporema* [41]. In another instance, Wennmann et al. (2015) employed two OB concentrations of AgseNPV-B equivalent to the LD10 and LD50 (determined at 7 dpi), mixed with a unique concentration of AgseGV equivalent to the LD50 (determined at 15 dpi) [34]. Cuartas et al. (2019) prepared mixtures of SfMNPV and SpfrGV by combining varying percentages of total OBs, with GV constituting the smallest proportion, from 2.5 to 10% of the mixture [24]. With a similar scheme, mixtures of SporNPV and SporGV were assayed on *Spodoptera ornithogalli* larvae [25].

### 3.3. Effects of Administering NPV and GV at Different Times

Combinations of NPVs and GVs were also tested by administering both viruses at separate times. Bird (1959) found that feeding CfGV granules 1 or 2 days before administering NPV polyhedra had an inhibitory effect on the development of polyhedrosis in the spruce budworm. However, by manipulating the times of virus feeding, distinct levels of double infections were observed, comprising blocks of adjacent cells infected with either NPV or GV. Individual cells infected with both viruses seemed to be an extremely rare event [26]. Lowe and Paschke inoculated NPVs 5–7 days after feeding granules, resulting in no development of polyhedrosis in *T. ni* [29]. Moreover, Hackett et al. (2000) found that feeding larvae with HearGV up to 36 h after HzSNPV ingestion led to the inhibition of NPV replication. This suggested that the interference observed was not solely a competition for host resources. Under this condition, HearGV outcompeted HzNPV, as microscopically examined cadavers revealed an abundance of GV granules rather than NPV polyhedra [18].

### 3.4. Selection of Larval Instar to Evaluate Synergism

The choice of larval instar to which the viral mixture is applied appears to affect the outcome of the interaction. However, most of the studies reviewed here focus on a single instar, and in general, the reasons for that selection are not detailed. Only a few studies have tested viral mixtures across two or more instars. Tanada highlighted the effectiveness of a mixed infection of PsunNPV + PsunGV vs. individual infections in the later developmental stages of the armyworm *P. unipuncta* [22]. According to his experience with *P. unipuncta*, “Fifth-instar larvae were found to be most suitable for tests on synergism because they were more resistant to virus infections than those in the first four instars, and hence demonstrated the synergistic association between the viruses much more distinctly” [22,81]. Moreover, Goto (1990) observed that XcenGV enhanced XcenNPV infection in the fifth-instar cutworms but not in the fourth-instar larvae [32]. Jeyarani (2010) evaluated the second, third, fourth, and fifth instars of *H. armigera* with different combinations of HearGV and HearNPV. Although they confirmed that the susceptibility of larvae to individual virus infections decreased with increasing instars, they did not observe an enhancement in any of the assayed instars [31]. In a study by Wennmann et al. (2015) conducted on neonate larvae of *A. segetum*, an additive effect was observed [34]. Considering the aforementioned studies, it raises the question of whether an enhancing effect might have been observed if older instars of *A. segetum* were employed.

### 3.5. Mathematical Determination of the Interaction Effect

It was not until 1968, when Lowe and Pashke introduced the bioassay technique and quantification of OBs [30], that data could be produced to quantitatively measure the effect of viral mixtures. However, even to this day, there is no unified criterion in the literature on how to measure the effect of baculovirus interaction on the host. For example, Lara-Reyna et al. (2003) analyzed the type of baculovirus interaction using different procedures, such as the ANOVA test for the LC50s, the Plackett and Hewlett joint-action rate test, and the Tammes–Bakuniak graphic method [39]. All these tests enable the differentiation between additive, synergistic, or antagonistic baculovirus interactions. The Tammes–Bakuniak graphic method quantitatively determines the type of interaction by measuring the deviation of the equitoxic line defined by the equation (z1/Z1) + (z2/Z2) = 1, where z1 and z2 are the quantities of both viruses in the mixture, and Z1 and Z2 are the doses of each virus that yield the same effect (e.g., LC50) when tested individually [82]. Therefore, the effect is called synergistic or antagonistic when the deviation from the equitoxic line is <1 or >1, respectively. They also used the joint action ratio R, which, according to the Plackett and Hewlett equation, is R = 1 [1 + (k + 1)1/2], where k is estimated from the equations V1 + kV1V2 + V2 = 1 and V1 = z1/Z1, and V2 = z2/Z2. The effect is considered additive, synergistic, or antagonistic when the result of the equation is R = 1, R > 1, or R < 1, respectively [83]. These methods are useful when the LD50 of both viruses can be determined, i.e., when both produce infection in the host. Thus, the use of this methodology is not viable to analyze baculovirus interactions where only one virus is infective.

An alternative method, based on mortality rates, can be used to determine the type of interaction in baculovirus mixtures [25]. The method, as described by Koppenhöfer and Kaya (1997) [16], compares the expected mortality of mixed infections with the observed mortalities recorded in bioassays. The following formula is applied: EM = M_NPV_ + M_GV_ (1 − M_NPV_), where M corresponds to the mortality caused by each virus alone, while EM is the expected mortality resulting from the combination of viruses. The calculated mortality (CM) in bioassays performed with viral mixtures is related to EM by chi-square value analysis (X^2^ = (CM − EM)^2^/EM). The X^2^ value is compared to the table value (X^2^ = 3.841) for 1 degree of freedom (dF) and α = 0.05. If the calculated X^2^ value is below 3.84, the virus interaction is categorized as additive. Conversely, values higher than 3.84 are associated with synergistic or antagonistic interactions. The difference in value (D) between CM and EM determines whether the virus interaction is synergistic (positive value) or antagonistic (negative value). In summary, the synergistic, additive, or antagonistic effect can be assigned based on quantitative analysis [16,25]. In order to exemplify this, we took results from the literature reviewed here and determined the kind of interaction using the method described above based on mortality rates (Table S2). Calculations were performed only where sufficient data was available, and the results are shown in Table 1. For example, Whitlock (1977) demonstrated that infections of *H. armigera* larvae with NPV and GV mixtures produced a viral effect described as “interference” [28]. Using the method mentioned above, this virus interaction should be classified as antagonistic (Table 1). Likewise, Bird (1959) analyzed the mortality rates of spruce budworm larvae by co-infecting them with CfMNPV and CfGV. The author’s results indicated that mortality registered in double infections is greater than in single virus infections, even though co-infected larval tissues analyzed under electron microscopy suggested NPV and GV interference [26]. Taking into account the mortality rates registered, the calculated X^2^ and D values indicate an antagonistic interaction (Table 1). In a recent study, Decker-Franco et al. (2021) compared the pathogenesis of mixed infections with AcMNPV and RanuNPV in *Rachiplusia nu* larvae. These authors deduced that co-infection did not produce a synergistic or antagonistic effect on the host [69]. In this case, we computed the X^2^ and D values, revealing an additive interaction (Table 1 and Table S2). Hence, the method described by Koppenhöfer and Kaya (1997) is a practical alternative to determining the type of interaction in mixed baculovirus infections. Furthermore, an examination of Table 1 allows us to observe a possible association between a decreased mean lethal time (LT50) and a synergistic interaction [37,41,42]. In the same way, some mixed infections reported as showing interference also showcased delayed host mortality compared to viruses applied separately [18,28,31]. In these cases, provided that mortalities were reported, we could recalculate the effects and classify them as antagonists.

When the enhancement effect is evident, another indicator is also used: the relative potency (RP). This concept was initially introduced when the GV was non-infective to the host and was used in combination with an NPV [37]. The RP is the ratio between the LD50 (also LC50 or ID50) of NPV alone and the LD50 of the NPV + GV viral mixture (Table 1). Based on this ratio, an enhancement index (log10) was also introduced [32,36]. The RP can be employed, either when the GV is infective to the host or not, to measure the level of enhancement produced by the addition of GV to NPV in viral mixtures [24,25]. It is worth noting that the term “enhancement” is usually associated with a synergistic effect, although this effect is not formally calculated in all studies.

### 3.6. Progeny Analysis and Diagnostics

In order to investigate whether larvae that had been inoculated with two viruses succumbed to NPV, GV, or a combination of both, early works diagnosed infected cadavers through light microscopy [32]. In cases where interference was reported, cells exhibiting mixed infections were rarely seen. Bird (1959) documented that double-infected *C. fumiferana* larvae showed different outputs: only polyhedrosis, only granulosis, or the presence of both polyhedra and granules in adjacent cells [26]. Similarly, Lowe (1968) reported different types of infection in adjacent cells of the fat body following double infection, but never found a single cell infected with both viruses [29].

Lara-Reyna et al. (2003) analyzed cadavers under the microscope after treatments with AcMNPV + TnSNPV and AcMNPV + TnGV, observing the dominance of AcMNPV OBs [39]. Similar results were obtained by Decker-Franco et al. (2021), in which mixed infection with AcMNPV and RanuNPV was confirmed using optical microscopy to examine larval tissues of *Rachiplusia nu* [69]. Nowadays, molecular techniques based on the polymerase chain reaction (PCR) permit the identification and quantification of different viruses coexisting within the same host. Studies performed by Wennmann et al. (2014) described the use of multiplex PCR and quantitative PCR (qPCR) for determining the AgseNPV-B/AgseGV ratio in samples of *A. segetum* larvae with mixed infections [83].

## 4. Discussion and Conclusions

Knowledge of in vivo baculovirus interactions offers the possibility of improving pest management strategies. A detailed review of studies on mixed baculovirus infections has enabled us to recognize different factors involved in the final effect on the host. Our extensive review revealed that mixed infections of NPV + GV are more intensively studied in comparison to NPV + NPV interaction studies. Regarding GV + GV mixtures, no study has been published describing natural co-infection produced by different GVs. However, several studies have described natural or experimental co-infections with mixtures of GV genotypic variants [84,85,86] or laboratory assays using closely related GVs on permissive hosts [87].

Our analysis of published studies reveals that the terminology used to describe viral interactions is associated with different classification criteria. For example, terms such as ‘interference’ or ‘neutralism’ refer to the consequences of the infection and/or replication of one virus due to the presence of another virus within the same host [18,28,42]. However, in several studies, the same terms have been applied to the effect of the virus interaction on the host itself. To clarify this concept, ‘interference’ refers to a situation where one virus hampers the replication of another virus, and ‘antagonism’ is the effect of the combination of two viruses on the host according to mortality rates. In this review, we propose to use the terms ‘synergistic’, ‘additive,’ or ‘antagonistic’ when referring to the effect on the host. This classification was used by Koppenhöfer and Kaya (1997) to describe the combined action of different agents (chemicals or biologicals) on insects. Furthermore, these authors determined the type of interaction using mathematical procedures based on observed and calculated mortality rates [16]. In our hands, in cases where some “interference” was reported, it could be interpreted as antagonistic or additive (Table 1), provided that there was enough data in the original report to calculate the effect. The proposed nomenclature proves valuable for virus interaction analysis because it can be used when the insect host is susceptible to one or both viruses in the mixture. On the other hand, the revision of baculovirus interaction experiments reveals that lethal time response analysis is a parameter to take into account from a biocontrol point of view. In most cases where the lethal time was evaluated and an enhancement or synergistic effect was observed, the viral mixture seemed to reduce the time to death (Table 1). Consistent with this observation, several reports demonstrated increased LT50s associated with antagonistic effects [28,31,33]. Therefore, a reduction in LT50 appears to be indicative of synergism.

The effect of baculovirus mixtures on the host depends on different factors such as virus inoculum amounts, host instar, host susceptibility, and the presence of infection-enhancing proteins associated with OBs. When both NPV and GV infect the same host, it appears that a low proportion of GV is associated with the synergistic effect, whereas when more GV is included, a neutral or antagonistic effect is promoted, as observed with XcenGV [32]. In the same way, SpfrGV had a synergistic effect when it was no more than 2.5% of a mixture with SfMNPV on *Spodoptera frugiperda* larvae. Also, it was found that virulence is reduced when the proportion of GV increases [24]. A recent study performed by Barrera et al. (2021) demonstrated that a viral mixture containing 97.5% of SporNPV and 2.5% of SporGV showed the highest insecticidal activity on the host [25]. On the contrary, in mixtures where a non-infective GV was used to enhance NPV virulence on the host, bioassays demonstrated that enhancement effects occurred with the highest GV concentrations evaluated [37,80]. Therefore, it is important to determine the best proportions of NPV and GV OBs to prevent antagonism and maximize the synergistic effect when applying mixtures as biological control agents. Another factor of consideration emerges in cases where the GV is not infective to the NPV target host: the selection of the amount of GV to incorporate into the mixture can be influenced by the potential impact of the GV on another lepidopteran host appearing simultaneously in the affected crop, as proposed by Biedma et al. (2015) [41].

Several reports indicate that the effects of mixed baculovirus infections can vary across different larval instars of the host. Initial studies with PsunNPV and PsunGV mixtures performed by Tanada showed a synergistic effect on the third to sixth instars of *Pseudaletia unipuncta* [22,23,53]. Different results were obtained in co-infections using HearNPV combined with HearGV on the second to fifth instars of *Helicoverpa armigera,* in which an antagonistic interaction was registered [31]. Goto et al. (1990) demonstrated different types of virus interaction using XcenNPV and XcenGV on fourth and fifth larval instars: in the fifth instar, a synergistic action was observed, while in the fourth instar, the co-infection resulted in a putative antagonistic effect (‘interference’) [32]. Thus, the synergistic effect appears to be more pronounced (or even observed) in later larval instars when the two viruses infect the host. This is in accordance with the fact that larvae are less susceptible to infection by NPV as they become older, and consequently, the enhancing effect of the GV is more visible in later instars [53]. However, a different situation was observed when the GV is not infective: Webb (2001) showed that the synergistic effect of HearGV on LdMNPV in *L. dispar* is lost in older instars [80]. Taken together, great variability can be observed in the viral interaction effect on different larval instars. Therefore, it is advisable to assay interaction effects in various instars to gain a more comprehensive understanding of what might happen in the field when applying a viral mixture.

An important issue extensively studied in baculovirus interactions refers to infection-enhancing proteins encoded by viral genomes. Enhancing effects are apparently confined to the NPV entrance because they are caused by enhancin-containing GVs/NPVs or by non-infective GV/NPVs (Table 1 and Table 2). It is worth noting that most of the GVs used in these works are type I GVs, the slow-killing type, and harbor one or more enhancin genes within their genomes (Table 1). The exceptions are EpapGV and SporGV, which are type II and type I granuloviruses, respectively, devoid of enhancin genes [25,88,89]. In the case of SpltGV, which synergized with HearNPV [42], there is no genome available to determine if it codes for *enhancin* genes. In cases where an enhancin-containing GV elicits interference, approaches such as virus inactivation or granule extract utilization could be viable alternatives to retain the enhancing effect while eliminating the interfering outcome. Furthermore, other proteins encoded in baculovirus genomes, such as ODV-E66 and chitinases, have shown potential for enhancing activities. These proteins were detected in the ODV and OB, respectively. However, their roles in baculovirus interactions remain to be studied. In this sense, proteomic approaches can shed light on which OB proteins are involved in the enhancing effect of some viruses. In line with this, EpapGV and SpfrGV proteomics added important information to the possible enhancer proteins involved in the synergistic effects observed for these viruses ([73], Masson et al., unpublished data).

Although in this review we do not analyze baculovirus covert infections, it is an important issue to consider in pest control strategies. Sublethal and latent infections produced by NPVs or GVs are frequently found in natural insect populations [9]. It has been reported that insects with sublethal infections are more susceptible to subsequent viral infections [90,91]. Thus, from a biocontrol perspective, covert infections may benefit pest control programs as lower quantities of OBs might be needed in field applications. However, in contrast to the potential benefits of covert infections, the literature reviewed here indicated that infections with NPVs in larvae previously infected by GV might reduce the effect of NPV [27]. Also, it is possible that treatment with GV in previously NPV-infected larvae abolishes NPV infection [18]. Therefore, it becomes necessary to study the results of mixed infections produced in insects with prior infections under field conditions to infer their possible implications for pest management [18].

Typically, commercial baculovirus formulations consist of OBs from a single baculoviral species [92,93]. Thus, these insecticides have a host range limited to one or a few insect pests. A potentially viable alternative is to prepare formulations containing a combination of different baculoviruses. This approach has the potential to expand the limited spectrum of baculovirus target pests. A notable example is the commercial biopesticide VPN ULTRA made in Guatemala by the company Agrícola El Sol which presents a combined formulation with the generalist AcMNPV and Spodoptera albula NPV baculovirus. This product is used to control a total of 7 different lepidopteran pests found in alfalfa and vegetable crops [93]. 

With a novel approach, recent studies describe an innovative technology for the development of baculovirus-based biopesticides. Several works have described the co-occlusion of baculovirus variants of different NPV baculovirus species under laboratory conditions [94]. The co-occluded variants of 

Chysodeixis chalcites nucleopolyhedrovirus (ChchNPV) obtained by cell culture selection resulted in an increased OB relative potency and a faster speed of kill. Similar results were observed with co-occluded mixtures of HearNPV in laboratory and field bioassays [95,96]. Additionally, the possibility of co-occluding different species of NPVs was confirmed by experimental larval inoculation with heterologous and homologous viruses. Thus, OBs with co-occluded AcMNPV + SfMNPV, AcMNPV + MbMNPV, SfMNPV + MbMNPV, and SfMNPV + SeMNPV were evaluated using insect bioassays [97]. This technology provides a new tool for the study of in vivo virus interaction and is a promising alternative to simultaneously control lepidopteran pests on particular crops.

From an insecticide perspective, synergism emerges as an ideal interaction between viruses included in a baculoviral formulation. Also, additive effects can be tolerated if the combination broadens the spectrum of pest targets that can be controlled under field conditions. However, in cases where antagonism is possible, the selection of viruses to combine needs to be considered cautiously, and proper assays should be performed to determine the final formulation. The use of mixed formulations with GV or NPV that harbor enhancin-like proteins and infect only their respective hosts is certainly of value if target insects appear simultaneously on the same crop [41]. On the other hand, the cost of baculovirus OB production is an important issue. Therefore, it is desirable to use the minimum doses of both viruses that provide the optimal mortality rate across most larval instars and the shortest lethal time under field conditions.

In this review, we analyze in vivo baculovirus interactions and their potential implications for pest control strategies. Combining baculovirus species from a biological control perspective requires conducting laboratory and field assays to determine the final effect on target insects. We conclude that the use of baculovirus-based formulations with NPV-GV or NPV-NPV compositions is an attractive approach to enhancing the insecticidal properties of one of the viruses in the mixture and achieving simultaneous pest control.

## Data Availability

Not applicable.

## Supplementary Materials

The following supporting information can be downloaded at: https://www.mdpi.com/article/10.3390/v15091838/s1, Table S1. Virus names and abbreviations; Table S2. Mathematical determination of interaction effects.

## Abbreviations

ODV	Occlusion-derived viruses
OB	Occlusion body
BV	Budded virus
NPV	Nucleopolyhedrovirus
GV	Granulovirus
MTD	Mean time to death
LD10	Lethal dose 10
LD25	Lethal dose 25
LD50	Lethal dose 50
LD95	Lethal dose 95
LC50	Lethal concentration 50
LT50	Lethal time 50
ID50	Infectious dose 50
dpi	Days post-infection
GVP	Granulovirus proteins
VEF	Viral enhancing factor
PM	Peritrophic membrane
CM	Calculated mortality
EM	Expected mortality
RP	Relative potency
PCR	Polymerase chain reaction
qPCR	Quantitative polymerase chain reaction
df	Degree of freedom
ANOVA	Analysis of variance
ND	Not determined
