# Supplementary material for: Effects of Mixed Baculovirus Infections in Biological Control: A Comprehensive Historical and Technical Analysis
Source: Viruses. 2023 Aug 30;15(9):1838. doi: 10.3390/v15091838 (PMC10534452; doi:10.3390/v15091838)
Supplement: Supplementary file 1 [file viruses-15-01838-s001.zip › viruses-2521516-supplementary Table S1.pdf]

Virus name	Abbrev.	Species (ICTV 2022)	Isolate	Accession (1)	Reference
Agrotis segetum nucleopolyhedrovirus B	AgseNPV-B	*Alphabaculovirus alteragsegetum*	English	KM10298	[34]
Bombyx mori nucleopolyhedrovirus	BmNPV	*Alphabaculovirus bomori*			[36]
Autographa californica multiple nucleopolyhedrovirus	AcMNPV	*Alphabaculovirus aucalifornicae*			[39]
Choristoneura fumiferana DEF multiple nucleopolyhedrovirus	CfDEFMNPV	*Alphabaculovirus alterchofumiferanae*	Ireland	AY327402	[64]
Anticarsia gemmatalis multiple nucleopolyhedrovirus	AgMNPV	*Alphabaculovirus angemmatalis*			[41]
Choristoneura fumiferana multiple nucleopolyhedrovirus	CfMNPV	*Alphabaculovirus chofumiferanae*	Ireland	NC_004778	[64]
Choristoneura fumiferana multiple nucleopolyhedrovirus	CfMNPV	*Alphabaculovirus chofumiferanae*			[26]
Chrysodeixis chalcites nucleopolyhedrovirus	ChchNPV	*Alphabaculovirus chrychalcites*			[95]
Helicoverpa armigera nucleopolyhedrovirus	HearNPV	*Alphabaculovirus helarmigerae*			[28, 31, 42]
Helicoverpa zea single nucleopolyhedrovirus	HzSNPV	--			[18, 66]
Lymantria dispar multiple nucleopolyhedrovirus	LdMNPV	*Alphabaculovirus lydisparis*			[37, 66]
Lymantria dispar multiple nucleopolyhedrovirus	LdMNPV	*Alphabaculovirus lydisparis*	LPD-226		[80]
Mamestra brassicae multiple nucleopolyhedrovirus	MbMNPV	*Alphabaculovirus mabrassicae*	T5		[43-47]
Pseudaletia unipuncta nucleopolyhedrovirus	PsunNPV	--			[22, 35, 36, 48, 49]
Rachiplusia nu nucleopolyhedrovirus	RanuNPV	*Alphabaculovirus ranus*	Argentinian		[67, 68]
Spodoptera exigua multiple nucleopolyhedrovirus	SeMNPV	*Alphabaculovirus spexiguae*			[66]
Spodoptera littoralis nucleopolyhedrovirus	SpliNPV	*Alphabaculovirus splittoralis*	Moroccan		[33]
Spodoptera litura nucleopolyhedrovirus	SpltNPV	*Alphabaculovirus spliturae*			[36, 40]
Spodoptera frugiperda multiple nucleopolyhedrovirus	SfMNPV	*Alphabaculovirus spofrugiperdae*	Col	KF891883	[24]
Spodoptera frugiperda multiple nucleopolyhedrovirus	SfMNPV	*Alphabaculovirus spofrugiperdae*			[66]
Spodoptera ornithogalli nucleopolyhedrovirus	SporNPV	--			[25]
Thysanoplusia orichalcea multicapsid nucleopolyhedrovirus	ThorMNPV	*Alphabaculovirus thorichlaceae*			[63]
Thysanoplusia orichalcea single nucleopolyhedrovirus	ThorSNPV	*Alphabaculovirus thorichlaceae*			[63]
Trichoplusia ni single nucleopolyhedrovirus	TnSNPV	*Alphabaculovirus trini*	Mexican		[39]
Xestia c-nigrum nucleopolyhedrovirus	XcenNPV	--			[32]
Achaea janata granulovirus	AjGV	--			[42]
Agrotis segetum granulovirus	AgseGV	*Betabaculovirus agsegetum*	DA	KR584663	[34]
Choristoneura fumiferana granulovirus	CfGV	*Betabaculovirus chofumiferanae*			[26]
Chillo infuscatellus granulovirus	CiGV	--			[42]
Cydia pomonella granulovirus	CpGV	*Betabaculovirus cypomonellae*			[70]

Epinotia aporema granulovirus	EpapGV	*Betabaculovirus epaporemae*	Argentinian	JN408834	[41]
Helicoverpa armigera granulovirus	HearGV	*Betabaculovirus helarmigerae*			[18, 28, 31, 37, 80]
Pseudaletia unipuncta granulovirus	PsunGV	*Betabaculovirus myunipunctae*			[22, 23, 35, 36, 53, 54]
Plutella xylostella granulovirus	PlxyGV	*Betabaculovirus pluxylostellae*			[42]
Spodoptera littoralis granulovirus	SpliGV	--			[33]
Spodoptera litura granulovirus	SpltGV	*Betabaculovirus spliturae*			[42]
Spodoptera frugiperda granulovirus	SpfrGV	*Betabaculovirus spofrugiperdae*	VG008	KM371112	[24]
Spodoptera ornithogalli granulovirus	SporGV	--			[25]
Trichoplusia ni granulovirus	TnGV	*Betabaculovirus trini*			[27, 39]
Xestia c-nigrum granulovirus	XecnGV	*Betabaculovirus xecnigri*	alpha4	AF162221	[32, 40, 43-47]

(1) Accesion numbers refer to genomic sequences of the isolates mentioned in the literature reviewed, if available.
