# Supplementary material for: Effects of Mixed Baculovirus Infections in Biological Control: A Comprehensive Historical and Technical Analysis
Source: Viruses. 2023 Aug 30;15(9):1838. doi: 10.3390/v15091838 (PMC10534452; doi:10.3390/v15091838)
Supplement: Supplementary file 1 [file viruses-15-01838-s001.zip › viruses-2521516-supplementary Table S2.pdf]

Table S2. Studies on NPV + GV combinations in larval hosts.

Reference	Host	Baculovirus Mixture	NPV	Infective	Mortality NPV	GV	Infective	Mortality GV	Mortality Mixture	X2 calculated	D	Interaction type calculated
[22]	*Pseudaletia unipuncta*	NPV+GV	PsunNPV	Yes	20 (3rd instar)	PsunGV	Yes	85	90	0,04	---	Additive
[22]	*Pseudaletia unipuncta*	NPV+GV	PsunNPV	Yes	35 (4th instar)	PsunGV	Yes	55	100	12,1	29,25	Synergistic
[22]	*Pseudaletia unipuncta*	NPV+GV	PsunNPV	Yes	16,1 (5th instar)	PsunGV	Yes	22,5	81,1	60,81	46,12	Synergistic
[22]	*Pseudaletia unipuncta*	NPV+GV	PsunNPV	Yes	3,4 (6th instar)	PsunGV	Yes	20	80	144	57	Synergistic
[26]	*Choristoneura fumiferana*	NPV+GV	CfMNPV	Yes	69,6	CfGV	Yes	64,4	83,6	38,38	-5,84	Antagonistic
[27]	*Trichoplusia ni*	NPV+GV	TnNPV	Yes	ND	TnGV	Yes	ND	ND	----	----	--------
[28]	*Heliotis armigera*	NPV+GV	HearNPV	Yes	58	HearGV	Yes	51,4	32,2	28,3	-47	Antagonistic
[80]	*Limantria dispar*	NPV+GV	LdMNPV (10exp11)	Yes	69 (2nd instar)	HearGV (1%)	No	0	87	4,69	18	Synergistic
[80]	*Limantria dispar*	NPV+GV	LdMNPV (10exp11)	Yes	69 (2nd instar)	HearGV (0.1%)	No	0	66	0,13	---	Additive
[80]	*Limantria dispar*	NPV+GV	LdMNPV (10exp10)	Yes	33 (2nd instar)	HearGV (1%)	No	0	62	25,48	29	Synergistic
[80]	*Limantria dispar*	NPV+GV	LdMNPV (10exp10)	Yes	33 (2nd instar)	HearGV (0.1%)	No	0	35	0,12	---	Additive
[80]	*Limantria dispar*	NPV+GV	LdMNPV (10exp11)	Yes	46 (3rd instar)	HearGV (1%)	No	0	85	33	39	Synergistic
[80]	*Limantria dispar*	NPV+GV	LdMNPV (10exp11)	Yes	46 (3rd instar)	HearGV (0.1%)	No	0	58	3,13	---	Additive
[80]	*Limantria dispar*	NPV+GV	LdMNPV (10exp10)	Yes	22 (3rd instar)	HearGV (1%)	No	0	47	28,4	25	Synergistic
[80]	*Limantria dispar*	NPV+GV	LdMNPV (10exp10)	Yes	22 (3rd instar)	HearGV (0.1%)	No	0	20	0,18	---	Additive
[80]	*Limantria dispar*	NPV+GV	LdMNPV (10exp11)	Yes	66 (4th instar)	HearGV (1%)	No	0	90	8,72	24	Synergistic
[80]	*Limantria dispar*	NPV+GV	LdMNPV (10exp11)	Yes	66 (4th instar)	HearGV (0.1%)	No	0	69	0,13	---	Additive
[80]	*Limantria dispar*	NPV+GV	LdMNPV (10exp10)	Yes	48(4th instar)	HearGV (1%)	No	0	52	0,33	---	Additive
[80]	*Limantria dispar*	NPV+GV	LdMNPV (10exp10)	Yes	48 (4th instar)	HearGV (0.1%)	No	0	35	3,52	---	Additive

[31]	*Helicoverpa armigera*	NPV+GV	HearNPV	Yes	57,8 (2nd instar)	HearGV	Yes	61,1 (2nd instar)	56,7 (2nd instar)	8,07	-26	Antagonistic
[31]	*Helicoverpa armigera*	NPV+GV	HearNPV	Yes	51,1 (3rd instar)	HearGV	Yes	59,8 (3rd instar)	58,9 (3rd instar)	5,72	-21	Antagonistic
[31]	*Helicoverpa armigera*	NPV+GV	HearNPV	Yes	54,5 (4th instar)	HearGV	Yes	56,7 (4th instar)	56,7 (4th instar)	6,93	-23	Antagonistic
[31]	*Helicoverpa armigera*	NPV+GV	HearNPV	Yes	55,6 (5th instar)	HearGV	Yes	45,5 (5th instar)	53,3 (5th instar)	6,67	-22	Antagonistic
[34]	*Agrotis segetum*	NPV+GV	AgseNPV-B	Yes	60,1	AgseGV	Yes	36,8	73,7	0,015	---	Additive
[41]	*Anticarsia gemmatalis*	NPV+GV	AgMNPV	Yes	41,7	EpapGV	No	0	80,6	36,2	39	Synergistic
[25]	*Spodoptera ornitogalli*	NPV+GV	SporMNPV	Yes	67	SporGV	Yes	6,66	98	12,06	28	Synergistic
**Reference**	**Host**	**Baculovirus Mixture**	**NPV 1**	**Infective**	**Mortality NPV 1**	**NPV 2**	**Infective**	**Mortality NPV 2**	**Mortality Mixture**	**X2 calculated**	**D**	**Interaction type**
[69]	*Rachiplusia nu*	NPV+NPV	AcMNPV	Yes	98,5	RanuNPV	Yes	91,9	100	0,001	---	Additive
[61]	*Trichoplusia nu*	NPV+NPV	AcMNPV	Yes	ND	TnSNPV	Yes	ND	ND	----	---	----
[63]	*Pseudoplusia includens*	NPV+NPV	ThorMNPV	Yes	ND	ThorSNPV	Yes	ND	ND	----	----	----

ND, not determined.
